# The role of naturally acquired antimalarial antibodies in subclinical *Plasmodium* spp. infection

**DOI:** 10.1002/JLB.5MR1021-537R

**Published:** 2022-01-20

**Authors:** Katherine O'Flaherty, Merryn Roe, Freya J.I. Fowkes

**Affiliations:** ^1^ Disease Elimination Program Burnet Institute for Medical Research and Public Health Melbourne Australia; ^2^ School of Public Health and Preventive Medicine Monash University Melbourne Australia; ^3^ Centre for Epidemiology and Biostatistics Melbourne School of Population and Global Health, The University of Melbourne Melbourne Australia; ^4^ Department of Infectious Disease Monash University Melbourne Australia

**Keywords:** Epidemiology, Malaria, Parasitic, Immune Response, Antibodies, Adaptive Immunity

## Abstract

Undetected subclinical *Plasmodium* spp. infections are a significant barrier to eliminating malaria. In malaria‐endemic areas, naturally acquired antimalarial antibodies develop with repeated infection. These antibodies can confer protection against the clinical manifestations of *Plasmodium* spp. infection in highly exposed populations, and several distinct functional antibody mechanisms have been defined in the clearance of *Plasmodium* parasites. However, the role of antimalarial antibodies during subclinical infection is less well defined. In this review, we examine the development and maintenance of antibody responses and the functional mechanisms associated with clinical protection, highlighted by epidemiological studies investigating the association between human immunity and detection of subclinical infection across various malaria transmission intensities. Understanding the development and role of the antimalarial antibody response during subclinical *Plasmodium* spp. infection will be essential to furthering novel interventions including vaccines and immunological biomarkers that can be utilized for malaria surveillance and ultimately progress malaria elimination.

## Graphical Abstract

This review examines the role of antibodies in sub‐clinical malaria, focusing on antibody development, and functional mechanisms, and epidemiological studies to inform malaria control interventions.

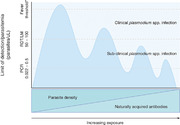

**Abbreviations**
AMA: apical membrane Ag
CSP: circumsporozoite protein
EBA: erythrocyte binding antigen
LM: light microscopy
MSP: merozoite surface protein
*P*.: *Plasmodium*
PCR: polymerase chain reaction
RDT: rapid diagnostic test
Spp.: species pluralis
WHO: World Health Organization

## 1 INTRODUCTION

Malaria control and elimination efforts have resulted in remarkable success over the past two decades, however, the latest World Health Organization (WHO) reports reveal that progress in malaria elimination has begun to plateau and that global cases increased in 2020.^1^ There are significant barriers to achieving malaria elimination. Most poignant is the ability to effectively detect and treat all *Plasmodium* spp. infections. Reported annual malaria cases do not represent the true number of *Plasmodium* spp. infections occurring worldwide, the majority of which are clinically silent, yet contribute significantly to ongoing transmission. Subclinical infection has been attributed to the non‐sterilizing nature of the naturally acquired antimalarial immune response in highly exposed populations. Such immunity is generally measured by the presence and magnitude of antibody responses in an individual or population. There are a number of studies investigating antigen‐specific antibody responses in protection from clinical manifestations of *Plasmodium* spp. infection, overwhelmingly they have found that increased antibody level or seropositivity, is associated with protection from clinical malaria. However, evidence for antibody‐mediated immunity in sub‐clinical *Plasmodium* spp. infection remains unclear. Here we review the literature examining naturally acquired antimalarial antibodies in sub‐clinical *Plasmodium* spp. infection in naturally exposed populations. Understanding the role of immunity in sub‐clinical *Plasmodium* spp. infection is crucial for our knowledge of malaria epidemiology and appropriate prevention, control, and surveillance activities, including novel vaccines and sero‐surveillance strategies.

## 2 MALARIA AND SUB‐CLINICAL *Plasmodium* SPP. INFECTION

Malaria is a vector‐borne disease caused by the protozoan parasite *Plasmodium* spp. and transmitted by *Anopheles* mosquitoes. There are five species of *Plasmodium* known to cause disease in humans (*P. falciparum, P. vivax, P. malariae, P. ovale*, and *P. knowlesi*), the two most prevalent and pathogenic being *P. falciparum* and *P. vivax*. Malaria is endemic to regions of sub‐Saharan Africa, South America, and the Asia Pacific. In high transmission settings in sub‐Saharan Africa, *P. falciparum* is the dominant species and children are at significant risk of serious illness compared to adults.^1^ In regions outside of Africa such as South America and South and Southeast Asia, the clinical incidence of malaria is comparatively low and transmission of several *Plasmodium* spp. occurs.^2^

Human infection begins with the inoculation of sporozoites by an infected female *Anopheles* mosquito as it takes a blood meal. Sporozoites (pre‐erythrocytic stages) then invade hepatocytes,^3^ and in the case of *P. vivax*, may remain there for many months or years in a dormant stage referred to as hypnozoites.^4^ Within the hepatocyte the parasite undergoes asexual replication, leading to the release of thousands of merozoites into the bloodstream.^3^ Within the erythrocyte merozoites mature into trophozoites, and then into merozoite‐filled schizonts that will burst, releasing between 16 and 32 new merozoites into the bloodstream that will go on to infect further erythrocytes. A proportion of blood‐stage parasites will commit to sexual differentiation and develop into transmissible gametocytes that are capable of being taken up by a feeding mosquito.^5^ The marked increase in parasite density from asexual blood proliferation, together with destruction of erythrocytes, is associated with the various clinical symptoms of malaria (fever, chills, fatigue, etc.).^6^

**FIGURE 1:**
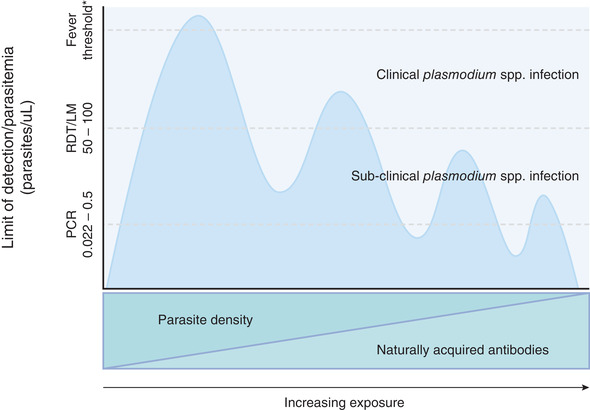
*Plasmodium* spp. parasite density dynamics, diagnostic detection limits, and presentation of infection with increasing exposure and development of antimalarial Abs. In malaria‐endemic regions, the development of antimalarial Abs is associated with protection from clinical malaria symptoms and increasing prevalence of subclinical infections that are often below the detection limits of conventional diagnostics (light microscopy [LM] and rapid diagnostic test [RDT]) and only detectable by polymerase chain reaction (PCR). ^*^Individual presentation of clinical malaria and parasite densities associated with the fever threshold vary. Created with BioRender.com

*Plasmodium* spp. infections that occur without the characteristic symptoms of malaria are referred to as subclinical (also termed asymptomatic). Though sometimes detectable by conventional diagnostics such as rapid diagnostic tests (RDTs) and light microscopy (LM), subclinical infections often consist of parasite densities below the detection limit of these diagnostic tools available in the field and clinical settings. In many malaria‐endemic settings, RDTs and LM have missed the majority of subclinical infections detected by more sensitive molecular diagnostics such as polymerase chain reaction (PCR), these are often referred to as sub‐microscopic infections that are in most but not all cases, also subclinical (Figure 1).

When compared with molecular detection methods, it is estimated that microscopy detects less than 50% of *P. falciparum* infections. This estimate varies significantly according to transmission intensity, with submicroscopic infections making up as much as 70% of all *Plasmodium* spp. infections in low transmission settings.^7^ Despite the low parasite density, sub‐clinical infections frequently transmit gametocytes to mosquitos,^8^, ^9^, ^10^ and despite this, this transmission being less effective when compared with high‐density infections,^10^, ^11^, ^12^, ^13^ sub‐clinical *Plasmodium* infections are likely to be responsible for a high proportion of mosquito infections due to their high prevalence.^14^ A recent systematic estimated that sub‐microscopic infections (many of which are also sub‐clinical) are the source of up to 68% of all human‐to‐mosquito infections.^7^ Given the importance of sub‐clinical infections to the ongoing transmission it is essential to understand the factors that underpin this infectious reservoir.

## 3 DEVELOPMENT OF ANTIMALARIAL IMMUNITY AND PROTECTION FROM CLINICAL ILLNESS

The epidemiology of clinical malaria can be explained by acquired immunity to *Plasmodium* spp., which develops over time and with repeated exposure to the parasite and can provide protection from high‐density parasitemia and the associated symptoms.^15^, ^16^ For this reason, children in high transmission settings who are yet to develop protective acquired immunity are at the greatest risk of suffering severe illness. In regions where malaria transmission is low and unstable, it is thought acquired immunity develops more slowly, subsequently, the burden of clinical disease is distributed more evenly across the age spectrum^15^ or in high‐risk individuals with increased exposure to malaria, for example, through occupational exposure.^17^ It is widely accepted that after repeated exposure, immunity functions to mediate low enough parasite densities to prevent clinical symptoms (Figure 1).

Naturally acquired Abs are among the most studied antimalarial‐immune response, and several of their functional mechanisms have been associated with protection from clinical falciparum malaria (reviewed in ref. 18). Induction of the antibody isotypes with functional capacity for complement deposition and Fc receptor interaction is dependent on Th2‐like T follicular helper cell subsets, as demonstrated in experimentally infected, previously malaria naïve individuals.^19^ The antimalarial antibody response consists of a predominantly IgG response; particularly the IgG1 and IgG3 subclasses that possess high‐affinity Fc receptors that can interact with complement and immune cells.^20^, ^21^ More recently, Ag‐specific IgM Abs have also been implicated in protection from clinical manifestations of *P. falciparum* infection.^22^ Antimalarial antibody functions include neutralization of invasion ligands,^23^ recruitment of complement factors,^24^ and enhanced phagocytosis either directly by monocytes via Fc receptor interaction or via complement fixation.^25^, ^26^, ^27^ Protection from clinical *P. falciparum* malaria has been associated with IgG and IgM specific for pre‐erythrocytic and blood‐stage targets. Humoral responses targeting the pre‐erythrocytic (sporozoite) stage of infection, namely the dominant circumsporozoite protein (CSP), can limit subsequent blood‐stage parasite density, and have been associated with delayed time to reinfection.^28^ Anti‐CSP IgG is associated with protection from clinical malaria,^28^, ^29^, ^30^ and has been shown to interact with complement protein C1q and mediate Fc‐receptor dependant functions such as phagocytosis by neutrophils and monocytes.^31^, ^32^

The blood stages, merozoites and the infected erythrocyte, are more widely studied in association with protection from clinical malaria, particularly during *P. falciparum* infection. The most studied targets include members of the merozoite surface protein (MSP) family including (MSP1, MSP2, MSP3), involved in attachment and re‐orientation of the merozoite, invasion ligands such as apical membrane antigen (AMA1), and erythrocyte‐binding Ags (EBA‐175, EBA‐140),^22^, ^33^, ^34^, ^35^, ^36^, ^37^, ^38^, ^39^ as well as parasite‐derived variant surface Ags expressed on the infected erythrocyte.^40^ Abs detected in response to specific merozoite Ags, whole merozoites, and the infected erythrocyte promote numerous effector functions associated with protection from clinical malaria symptoms and prospective risk of infection. These include interaction with cellular Fc receptors to enhance parasite uptake by phagocytic cells including neutrophils and promoting the release of pro‐inflammatory cytokines and reactive oxygen species leading to broader immune activation,^25^, ^26^, ^41^, ^42^, ^43^, ^44^, ^45^ fixation of complement factors, and activation of the classical complement cascade by parasite‐specific Abs and deposition of the membrane attack complex leading to parasite lysis,^24^, ^46^ and inhibition of parasite invasion through binding and neutralization of invasion ligands or inhibition of schizont rupture.^36^, ^47^, ^48^ Notably, fewer Ab‐dependant protective mechanisms have been identified for non‐falciparum *Plasmodium* spp. This includes for *P. vivax* infection where several Ag‐specific Ab responses have been identified but few have been associated with clinical protection (reviewed in ref. 49). Ultimately, the functional mechanisms against these targets slow parasite multiplication and limit blood‐stage parasitemia, preventing clinical symptoms associated with high parasite density. Despite strong evidence and defined mechanisms for a protective role of Abs in ameliorating clinical disease, the role of these mechanisms in perpetuating subclinical *Plasmodium* spp. infection and conversely how subclinical infection maintains antibody responses is less clear.

## 4 MAINTENANCE OF ANTIMALARIAL ANTIBODIES AND SUB‐CLINICAL *Plasmodium* SPP. INFECTION

The persistence of subclinical infections is generally accepted to be a consequence of frequent exposure to the parasite and the acquisition of nonsterilizing immunity, controlling parasite density below a clinical threshold. The persistence of anti‐merozoite IgG responses associated with circulating memory B‐cells has been shown for several years following the interruption of *P. falciparum* transmission.^50^ Furthermore, the half‐life of the Abs measured in response to merozoite Ags ranges between several years to life‐long persistence,^50^, ^51^, ^52^ demonstrating that long‐lived antimalarial Ab responses can be generated. However, differential declines in Abs promoting anti‐parasite functions such as complement deposition and opsonic phagocytosis compared to total Ab titers have been observed.^53^ There are, however, some discrepancies in the literature regarding the longevity of the antimalarial humoral response, likely due to the great heterogeneity in malaria epidemiology and study design in the populations investigated. Modeling of the estimated half‐life of Ab‐secreting cells using antibody kinetic data from endemic populations in the African region demonstrates that the antimalarial response is short‐lived without frequent re‐exposure.^54^, ^55^ Development of long‐lived memory B‐cell responses may be dysfunctional in malaria infection, with several studies observing the expansion of mostly short‐lived plasma cells following acute infection (reviewed in ref. 56). These short‐lived responses may be sufficient to sustain clinical immunity in regions with intense malaria transmission. However, the large proportion of subclinical infections in low transmission settings challenges current thinking about the development and maintenance of antimalarial immune responses as the transmission is mostly low and exposure is assumed to be infrequent.

The development of robust Ab responses in low transmission settings may require fewer exposures due to the lower number of circulating strains, and incident infections have recently been shown to be more likely to occur when there is the introduction of new strains.^57^ It has been proposed that in areas of low transmission intensity there are hot spots where a minority of residents are intensely exposed and develop robust immunity, allowing subclinical infection to persist in those individuals in an area that would otherwise be low transmission and hence its residents would have low levels of immunity.^58^ Low‐density parasitemia may be responsible for the maintenance of immunity associated with clinical protection in low transmission settings, rather than immunity driving the persistence of submicroscopic *Plasmodium* infection. The underlying subclinical infection itself has been implicated in protection from future incident malaria,^59^, ^60^ and hypothesized to be necessary for maintaining clinical immunity.^61^ This is consistent with observations of Ab boosting during infection with *Plasmodium* spp.^62^ However, current evidence implicating low‐density parasitemia in the maintenance of antimalarial immunity is conflicted, with some studies finding subclinical infections can precede clinical disease, and is more likely to occur where people are at greater risk of infection.^29^, ^62^ Further, Ab maintenance has been shown to occur at similar rates in children treated for subclinical infection and their uninfected counterparts, and treatment of underlying subclinical infection was not associated with prospective risk of *P. falciparum* infection.^63^

Parasite factors that explain the carriage of subclinical *Plasmodium* spp. infection have also been identified that are independent of acquired antimalarial immunity. Recent reports of replicating *P. falciparum* and *P. vivax* parasites accumulated within the spleen of splenectomized patients with no peripheral *Plasmodium* spp. infection provided evidence of a hidden parasite biomass.^64^, ^65^ Furthermore, recent studies conducted in Malian children in the context of highly seasonal and low transmission settings where the parasite may need to adapt to a low frequency of competent vectors demonstrated transcriptomic differences in *P. falciparum* parasites detected in the dry compared to wet (high‐transmission) season, accounting for longer circulation of infected erythrocytes and increased splenic clearance.^66^, ^67^ The authors hypothesize that these differences contribute to the reduced parasite densities observed during the dry season, allowing the parasite to avoid immune‐mediated clearance and persist until transmission conditions become more favorable. However, subclinically infected children did exhibit increased *P. falciparum*‐specific IgG responses and memory B cell activity compared to uninfected children, indicating that some preexisting exposure is associated with carriage of subclinical infection in highly seasonal transmission settings.^67^

Whether the development of protective Ab responses is responsible for the persistence of a low‐density infection, or if chronic low‐density infections are responsible for the maintenance of antimalarial antibodies remains unclear. Acquired antimalarial immunity is not generally thought to be sterilizing, and as discussed, chronic reservoirs of subclinical infection in intensely exposed populations are thought to be maintained by acquired immunity. Recent evidence suggests, however, that peripheral subclinical infections may not be as chronic as once thought, and that subclinical *P. falciparum* infections are regularly cleared in the absence of treatment. In high transmission settings, participants regularly clear and become infected with new parasite strains, presenting as a single infection over a long period of time unless molecularly genotyped.^63^ Further, recent intense monitoring of ultra‐low‐density infections in Vietnam and Cambodia revealed that the parasite density of many *P. falciparum* and *P. vivax* infections oscillates frequently and that many infections are spontaneously cleared in a matter of months.^68^, ^69^ Few studies have attempted to directly quantify the role of immunity in the spontaneous clearance of subclinical *Plasmodium* spp. infection. Recent studies have demonstrated an association between Abs and protection from PCR‐detectable subclinical infection.^70^, ^71^ However, these findings have been reported mostly in serial cross‐sectional surveys, which may fail to capture individual infection dynamics.

## 5 HUMORAL IMMUNITY AND THE PREVALENCE OF SUB‐CLINICAL *Plasmodium spp*. INFECTION

Evidence that Ag‐specific Ab responses are implicated in subclinical *Plasmodium* spp. infection comes from large‐scale serological surveys in malaria‐endemic populations. In many cross‐sectional surveys, antimalarial Abs are found at greater levels or in a greater proportion of subclinically infected individuals compared to uninfected community members for both *P. falciparum* and *P. vivax* infection across a range of transmission intensities (summarized in Table 1). Overwhelmingly, these studies have found seroprevalence and/or higher levels of antibodies and increased prevalence of subclinical infection, despite a variety of different parasite targets investigated.^61^, ^72^, ^73^, ^74^, ^75^, ^76^, ^77^, ^78^, ^79^ Although, like investigations of the role of acquired antimalarial Abs and protection from clinical manifestations of *Plasmodium* spp. infections, many studies have focused on sporozoite and merozoite‐specific responses with a lack of investigations into the role of Abs in response to the infected erythrocyte (Table 1). Collectively, the findings indicate that in many malaria‐endemic populations, Abs are biomarkers of underlying or recent *Plasmodium* spp. infection. Indeed, Abs can predict hot spots of infection in discrete regions,^80^, ^81^ and are being pursued as surveillance tools in many settings for detection of subclinical reservoirs.^82^ However, several other cross‐sectional studies investigating the same common blood‐stage antigenic targets have found little or no differences in the overall prevalence and breadth of antimalarial IgG between individuals with and without subclinical infection.^70^, ^83^, ^84^, ^85^ Many studies assessing Ab responses and the occurrence of subclinical infection performed to date have not been designed with the primary goal of assessing this association and are frequently performed as secondary outcomes in field surveys designed to assess some other primary outcome. The challenge of all serological surveys in malaria is the confounding effect of prior exposure, which in the vast majority of studies cannot be quantified and likely differs significantly between exposed populations. This caveat is particularly problematic in cross‐sectional surveys, where it is impossible to know when the last boosting event occurred, and how the development and kinetics of that response will change the association over time. Further, in regions that have recently transitioned from high/moderate to low transmission where a large proportion of a surveyed population may still be reflected as high responders or as seropositive.^71^ The findings of such investigations may provide interesting insight, however, large, observational studies using highly sensitive molecular diagnoses with frequent sampling among returning participants provide the most robust data for assessing associations between acquired immunity and subclinical infection.

**TABLE 1:** Cross‐sectional studies investigating antibodies in subclinical *Plasmodium* spp. infection

Study area, year	*Plasmodium* spp. (detection method)	Antibody response (antigen/s)	Summary
Low transmission settings			
Thailand, 2015^73^	*Pf, Pv* (PCR)	IgG response to 281 *Pf* and 177 *Pv* antigens (multiple life‐cycle stages)	IgG levels higher in sub‐clinical v. uninfected
Thailand, 2016^74^	*Pf, Pv, Po, Pm* (PCR)	IgG response to 184 *Pf* and 142 *Pv* antigens	IgG levels higher in sub‐clinical v. uninfected
Thailand, 2017^75^	*Pv* (PCR)	IgG detected on micro‐array to multiple blood stage antigens	IgG levels higher in sub‐clinical v. uninfected
Thailand, 2020^77^	*Pv* (PCR)	Anti‐merozoite IgG (*Pv*RBP2‐P1)	IgG levels higher in sub‐clinical v. uninfected
Myanmar, 2021^78^	*Pf, Pv* (PCR)	Anti‐merozoite IgG (*Pf/Pv*MSP1‐19 and AMA1)	IgG levels higher in sub‐clinical v. uninfected
Indonesia, 2021^83^	*Pv* (LM)	Anti‐merozoite IgG (*Pv*DPB)	IgG similar in infected v. uninfected
Brazil, 2010^85^	*Pv* (PCR)	Anti‐merozoite IgG (*Pv*MSP1)	IgG levels similar in infected v. uninfected
Brazil, 2013^79^	*Pv* (PCR)	Anti‐merozoite IgG and IgG1–4 (*Pv*MSP1)	IgG and IgG2/3 levels higher in sub‐clinical v. uninfected

Moderate to High Transmission Settings			
PNG, 2010^85^	*Pv* (PCR)	Anti‐merozoite IgG (*Pv*MSP1)	IgG levels similar in infected v. uninfected
Tanzania, 2009^72^	*Pf* (PCR)	Anti‐merozoite IgG (*Pf*MSP1, MSP2, AMA1)	IgG levels higher in sub‐microscopic v. uninfected
Uganda, 2013^61^	*Pf* (LM and PCR)	Anti‐sporozoite and merozoite IgG (*Pf*CSP, AMA1, MSP2, MSP1‐19)	IgG levels higher in sub‐microscopic v. uninfected
Kenya, 2015^84^	*Pf* (LM)	Anti‐sporozoite and merozoite IgG and IgG1–4 (*Pf*CSP, LSA‐1, TRAP, AMA1, EBA175, MSP1)	IgG1/3 levels higher in regions of stable malaria transmission, but similar in infected v. uninfected individuals
Gabon, 2017^70^	*Pf* (PCR)	Anti‐merozoite IgG (*Pf*113, AMA1, EBA175, Rh5)	IgG seropositivity not associated with sub‐clinical infection and greater in uninfected
Ghana, 2018^76^	*Pf* (LM)	Anti‐merozoite IgG (*Pf*EBA175‐RIII‐V)	IgG seroprevalence greater in regions with high prevalence of sub‐clinical infections

Abbreviations: LM, light microscopy; PCR, polymerase chain reaction.

Longitudinal studies can provide stronger evidence of the role of antibodies in subclinical *Plasmodium spp*. infection. Recent longitudinal investigations in naturally exposed populations, some implementing highly sensitive molecular diagnostics, have found antibody seroprevalence is associated with protection from clinical malaria and thus, an increased likelihood of subclinical parasitemia to some but not all parasite targets (Table 2).^86^, ^87^, ^88^, ^89^ Further, Ab levels in chronically infected individuals are observed at greater levels than those who will go on to develop an incident case of clinical malaria (Table 2).^14^, ^90^ Malaria transmission intensity is generally high, although heterogeneous throughout regions of sub‐Saharan Africa, but unlikely to reflect the transmission dynamics and acquisition of immunity in other malaria‐endemic settings where exposure may be less frequent, but the prevalence of sub‐clinical *Plasmodium* spp. infection is significant.

**TABLE 2:** Longitudinal studies investigating antibodies in subclinical *Plasmodium* spp. infection

Study area, year	*Plasmodium* spp. (detection method)	Antibody response (antigen/s)	Summary
Moderate to High Transmission Settings			
PNG, 2014^88^	*Pf* (LM and PCR)	Anti‐merozoite IgG (Rh5)	IgG was not associated with the time to a PCR detectable infection
Kenya, 2009^90^	*Pf* (LM)	Anti‐infected erythrocyte IgG (VSA)	Seroprevalence greater in uninfected v. clinically and sub‐clinically infected, seropositive children more likely to acquire a sub‐clinical v. clinical infection
Gabon, 2015^87^	*Pf* (LM)	Anti‐sporozoite, merozoite and infected erythrocyte IgG (CSP, AMA1, MSP1, MSP2, MSP3, *Pf*EMP1)	IgG levels higher in sub‐clinical v. clinically infected and uninfected, breadth of antibody response greater in clinically infected
Ghana, 2018^89^	*Pf* (LM)	Anti‐merozoite and gametocyte IgG (MSP3, s230)	IgG seroprevalence greater in regions with high prevalence of sub‐clinical infections
The Gambia, 2020^86^	*Pf* (PCR)	Anti‐merozoite, infected erythrocyte and gametocyte IgG (sHSP40.Ag1, GEXP18, MSP1‐19, AMA1, GLURP.R2, Etramp5.Ag1, Etramp4.Ag2, Hyp2)	IgG associated with sub‐clinical infection.
Burkina Faso, 2021^14^	*Pf* (PCR)	Anti‐sporozoite, merozoite and gametocyte IgG	IgG levels higher in chronic sub‐clinical infection v. those that develop a clinical infection

Abbreviations: LM, light microscopy, PCR, polymerase chain reaction.

## 6 CONCLUDING REMARKS

Furthering our knowledge of antimalarial immunity and the natural course of subclinical *Plasmodium* spp. infections will advance our understanding of the epidemiology of malaria. To progress this field, identifying the functional immune mechanisms associated with both chronicity and clearance of subclinical infection is required, as are well‐designed, longitudinal serological surveys with highly sensitive molecular diagnostics and inclusion of diverse malaria‐endemic populations. Most evidence to date has focused on the response to merozoite‐specific IgG responses, and future inclusion of a greater diversity of parasite targets including variant surface Ags may provide further insight into the role of Ab responses in the prevalence and maintenance of subclinical *Plasmodium* spp. infection. Additionally, a greater focus on non‐falciparum *Plasmodium* spp. infection is required, particularly for *P. vivax* where protective functional mechanisms are yet to be elucidated. Knowledge gained from these in‐depth investigations will be vital to the development of malaria elimination tools, including novel vaccine targets and sero‐surveillance markers.

## AUTHORSHIP

K.O. and M.S.R. performed the literature searches. K.O. wrote the first draft of the manuscript. M.S.R. and F.J.I.F. contributed to the writing of the manuscript. F.J.I.F. conceived the manuscript. All authors agreed to the final submission of the manuscript.

## DISCLOSURE

The authors declare no conflicts of interest.
